# Calponin 1 inhibits agonist‐induced ERK activation and decreases calcium sensitization in vascular smooth muscle

**DOI:** 10.1111/jcmm.18025

**Published:** 2023-12-26

**Authors:** Lova Prasadareddy Kajuluri, Qing Rex Lyu, Jaser Doja, Ajay Kumar, Michael P. Wilson, Samantha R. Sgrizzi, Elika Rezaeimanesh, Joseph M. Miano, Kathleen G. Morgan

**Affiliations:** ^1^ Vascular Biology Laboratory, Department of Health Sciences Boston University Boston Massachusetts USA; ^2^ Vascular Biology Center Medical College of Georgia at Augusta University Augusta Georgia USA; ^3^ Medical Research Center Chongqing General Hospital Chongqing China; ^4^ University of Rochester Medical Center Rochester New York USA; ^5^ Present address: Cardiovascular Research Center Massachusetts General Hospital Charlestown Massachusetts USA

**Keywords:** arterial stiffness, calcium sensitization, calponin, CRISPR, ERK, smooth muscle

## Abstract

Smooth muscle cell (SMC) contraction and vascular tone are modulated by phosphorylation and multiple modifications of the thick filament, and thin filament regulation of SMC contraction has been reported to involve extracellular regulated kinase (ERK). Previous studies in ferrets suggest that the actin‐binding protein, calponin 1 (CNN1), acts as a scaffold linking protein kinase C (PKC), Raf, MEK and ERK, promoting PKC‐dependent ERK activation. To gain further insight into this function of CNN1 in ERK activation and the regulation of SMC contractility in mice, we generated a novel *Calponin 1* knockout mouse (*Cnn1* KO) by a single base substitution in an intronic CArG box that preferentially abolishes expression of CNN1 in vascular SMCs. Using this new *Cnn1* KO mouse, we show that ablation of CNN1 has two effects, depending on the cytosolic free calcium level: (1) in the presence of elevated intracellular calcium caused by agonist stimulation, *Cnn1* KO mice display a reduced amplitude of stress and stiffness but an increase in agonist‐induced ERK activation; and (2) during intracellular calcium depletion, in the presence of an agonist, *Cnn1* KO mice exhibit increased duration of SM tone maintenance. Together, these results suggest that CNN1 plays an important and complex modulatory role in SMC contractile tone amplitude and maintenance.

## 1 INTRODUCTION

Calponin was first isolated from chicken gizzard and bovine aorta and was found to interact with F‐actin, calmodulin and tropomyosin.^1^, ^2^ Three isoforms of calponin were subsequently discovered in mammals: calponin 1 (basic calponin, CNN1), calponin 2 (neutral calponin, CNN2) and calponin 3 (acidic calponin, CNN3).^3^, ^4^, ^5^, ^6^ *Cnn1* mRNA is expressed transiently in the developing heart, but then is largely restricted to SMC in adult tissues.^7^ The other two isoforms are expressed more broadly in nonmuscle cells.^8^ *Cnn1* mRNA expression was shown to change when SMCs undergo a phenotypic switch with higher *Cnn1* levels in the differentiated phenotype than the proliferative phenotype.^9^ However, what role CNN1 protein plays in mediating SMC differentiation is unknown. In addition, the functional significance of CNN1 in differentiated vascular smooth muscle cells (VSMCs) is incompletely understood.

CNN1 has long been reported to regulate SMC contraction. Previous in vitro studies showed that CNN1 inhibits SMC contraction by preventing actin‐activated Mg^+2^‐ATPase activity of myosin.^10^ Other studies reported that lack of CNN1 reduced smooth muscle cell sensitivity to agonist stimulation.^11^, ^12^ In a recent study by Feng et al, depletion of CNN1 was shown to reduce maximal force production induced by norepinephrine, and *Cnn1* knockout aortas undergo faster relaxation after removal of KCl depolarizing solutions.^13^

Previous studies from our laboratory reported that CNN1 undergoes agonist‐induced translocation from central actin filaments to the cortical region of differentiated VSMCs.^14^ In addition, it has been shown that CNN1 interacts with both ERK (MAP kinase) and PKC and aids in ERK translocation to the cortical region of the VSMC where ERK gets activated.^15^ Nevertheless, the impact of CNN1 in mediating ERK activation remains unclear.

In the present study, we aimed to gain insight into the role of CNN1 in ERK activation and the regulation of VSMC contraction and tone. Based on an original report demonstrating the key role of an intronic serum response factor‐binding element called a CArG box,^16^ we generated a new *Cnn1* knockout mouse by introducing a single base substitution in the CArG box. The results from the current study show that in the presence of an agonist and physiologic levels of Ca^+2^, CNN1 promotes tone amplitude but inhibits ERK activation. However, in the absence of Ca^+2^, CNN1 inhibits prolonged tone maintenance, pointing to two different roles of CNN1 in different signalling pathways.

## 2 MATERIALS AND METHODS

### 2.1 Reagents

All chemicals used in this study were of molecular biology grade and are purchased from either Millipore SIGMA or Thermo Fischer Scientific. Antibodies for p44/42 ERK (cat‐4696S), Phospho p44/42 ERK (cat‐9101S) and Phospho MLC‐20 (cat‐3671S) were purchased from Cell Signaling Technology. The CNN1 (cat‐C2687) and GAPDH (cat‐G9545) antibodies were purchased from Millipore SIGMA. The CNN2 antibody was a gift from Dr. J.P Jin. CNN3 (cat‐sc‐271188) antibodies were purchased from Santacruz Inc. Phospho MYPT1‐T850 (cat‐AP0916) antibodies were purchased from ABclonal, and Phospho CPI17‐T38 (PA5‐36842) antibodies were purchased from Thermo Fischer Scientific. Vinculin antibody was purchased from SIGMA (cat‐V9131). The Nuc blue stain was purchased from invitrogen (cat‐R37605). Blocking buffer and secondary antibodies were purchased from Li‐COR. 5X protein loading dye was prepared as reported elsewhere.

### 2.2 Animals and aorta preparation

Animals were maintained in Boston University, and Augusta University according to the NIH guide for the care and use of laboratory animals. All procedures were performed in accordance with approved Institutional Animal Care and Use Committee protocols at Boston University (#201900004) and Augusta University (#2019–1000). Mouse aortas were quickly collected after death by isoflurane inhalation and placed in Krebs solution (120 mM NaCl, 5.9 mM KCl, 11.5 mM Dextrose, 1.2 mM NaH2PO_4_.H_2_O, 1.2 mM MgCl_2_.6H_2_O and 2.5 mM CaCl_2_) on ice.

### 2.3 Generation of *Cnn1* knockout mice

As a first step, we performed a competitive electromobility shift assay (EMSA) as described^16^ using a 32P‐labelled single strand deoxyoligonucleotide (ssODN) probe of sequence agccgccgcgccttataaggcggccttggg (Coralville, IA) containing a serum response factor (SRF) binding CArG box (underlined sequence) located in the first intron of *Cnn1*. This intronic CArG box was shown previously to be important for in vitro transcriptional activation of the *Cnn1* promoter.^16^ The above radiolabelled probe was combined with in vitro translated SRF in the absence or presence of excess unlabelled ‘cold’ ssODNs carrying single base substitutions across the labelled probe, and the nucleoprotein complexes were resolved in a nondenaturing 6% polyacrylamide gel. The gel was vacuum dried to Whatmann filter paper and exposed to x‐ray film at −80 C.

3‐component CRISPR^17^ was used to generate a novel mouse carrying a single C > G transversion in the *Cnn1* intronic CArG box. Briefly, a single guide RNA (Synthego) used previously to generate a more aggressive CArG mutation^18^ was combined with Cas9 protein (Integrated DNA Technologies) in a ribonuceloprotein complex (RNP). The RNP was incubated at room temperature for 5 min and then mixed with a ssODN containing the C > G transversion (cagagactgatggcagcgccgccccctccccccagcgccggccccagagtcgcagaggagccgccgcg **g** cttataaggcggccttgggcagcccgggcccgcgctatataagggccggtttgctttataaagccgggc; single base substitution bold; Integrated DNA Technologies). The RNP/ssODN mix was microinjected into zygotes (strain C57BL/6J) at a ratio of 3 pmol sgRNA: 3 pmol Cas9: 11 pmol ssODN in nuclease‐free M2 microinjection buffer (Sigma) and viable 2‐cell stage embryos were transferred to pseudopregnant ICR mice. Pups were genotyped by *SacII* digestion of a PCR product generated with primers flanking the edited allele (forward primer, 5’‐agggaacactgaggcactttctttccag‐3′; reverse primer, 5′‐ tagcttcagctcttatcccatcttcc‐3′). Founder mice were bred to C57BL/6J mice for germline transmission of the targeted allele, and Sanger sequencing confirmed sequence fidelity across the PCR product amplified with the aforementioned primers. Heterozygous intercrosses generated mice wild‐type or homozygous for the C > G transversion. There were no off‐targeting events in linkage disequilibrium with *Cnn1* based on the CRISPOR algorithm.^19^

### 2.4 Quantitative RT‐PCR

Tissues of different *Cnn1* genotypes were isolated and snap frozen in liquid nitrogen, homogenized with an automated Bullet machine (Next Advance), and total RNA isolated with TRIzol™ (Thermo Fisher Scientific). Expression analysis was conducted with a Bio‐Rad CFX96 real‐time qPCR system using the delta CT method. Primer sequences were *Cnn1* (forward: TCATCTGCACCTCTGCTTTG; reverse: GGGCCAGCTTGTTCTTTACT), *Actb* (forward: GAGGTATCCTGACCCTGAAGTA; reverse: CACACGCAGCTCATTGTAGA).

### 2.5 Bulk RNA‐seq

Total RNA from wild‐type and *Cnn1* KO mouse aorta (*n* = 3/genotype), less adventitia and endothelium, was isolated with RNeasy kit and submitted to the University of Rochester Genomics Research Center for RNA quality, library construction and sequencing with an llumina NextSeq 550 sequencer (San Diego, CA) at a sequencing depth of 70 million reads per replicate as described previously.^20^ PCA and volcano plots were generated using DESeq2 in RStudio. In brief, a counts matrix was generated from Fastq files and used as input for DEseq2 normalization and differential expression analysis. PCA plots and volcano plots were visualized from DEG‐ranked lists using ggplot with a Log_2_FC cutoff of 1 and a padj value cutoff of 0.01. Raw sequence data and expression changes were deposited at the Gene Expression Omnibus (GSE #239476).

### 2.6 Phenylephrine‐induced stress and stiffness

The thoracic aortas of young adult (4–5 months), wild‐type (WT) and *Cnn1* KO mice were excised after euthanization by isoflurane inhalation. Aortas were placed in Krebs solution (120 mM NaCl, 5.9 mM KCl, 11.5 mM Dextrose, 1.2 mM NaH2PO_4_.H_2_O, 1.2 mM MgCl_2_.6H_2_O and 2.5 mM CaCl_2_) on ice. The outer fatty layer was carefully removed in a sylgard dissection chamber containing Krebs solution supplied with 95% O_2_ and 5% CO_2_. Aortic rings of 0.5 mm were cut and placed in Eppendorf tubes containing Krebs solution with added Nuc‐blue cell‐permeant stain to visualize the nucleus of VSMCs for calculation of medial layer thickness. The thoracic aorta was cut into two 4–5 mm long strips followed by measuring their diameter under a dissection microscope. Each strip of the aorta was placed in an organ bath containing 50 mL of Krebs solution with 100 μM N(gamma)‐nitro‐L‐arginine methyl ester (L‐NAME). The temperature of the solution was maintained at 37°C using a circulating water bath and supplied with 95% O_2_ and 5% CO_2_. Aortic strips were stretched to 80% of the initial length (optimal length for force production) and allowed to equilibrate for 30–60 min. After equilibration, the standard Krebs solution was replaced with a depolarizing 51 mM KCl Krebs solution (30 mM NaCl, 51 mM KCl, 11.5 mM Dextrose, 1.2 mM NaH2PO_4_.H_2_O, 1.2 mM MgCl_2_.6H_2_O and 2.5 mM CaCl_2_) to check the viability of the tissues. After 15 min, the KCl Krebs solution was replaced with standard Krebs solution, and the muscle was allowed to relax for 30 min. After relaxation, the muscle was incubated with 10 μM phenylephrine‐Krebs solution to assay the SMC contraction amplitude. Stiffness was calculated using high‐frequency low amplitude (HFLA) length oscillations as previously described.^21^ After myography data acquisition, the tissues were snap frozen in ice‐cold (‐80°C) acetone‐dithiothreitol (DTT)‐trichloroacetic acid (TCA) solution, and samples were immediately placed in a −80°C freezer for further analysis.

### 2.7 Ca‐independent smooth muscle tone

To assess the Ca‐independent smooth muscle tone in WT and *Cnn1* KO, aortas were stretched to 80% of the initial length and allowed to equilibrate for 1 h in the Krebs solution. After equilibration, aortas were initially stimulated with 51 mM KCl Krebs solution for 15 min to check the viability of the tissue. After 15 min, the KCl Krebs solution was replaced by Krebs solution, and the tissues were allowed to relax for 30 min before treatment with 10 μM phenylephrine‐Krebs solution. After 15 min of phenylephrine treatment, smooth muscle tone was assessed by gradually reducing the calcium concentration in the buffer by adding EGTA to a final concentration of 3 mM. For the analysis of molecular mechanisms responsible for tone maintenance, aortas were quick‐frozen in a DTT‐TCA‐acetone solution under the following conditions: (1) when the aortas were maintaining tone, or (2) when the aortas reached the relaxed baseline. Once quickly frozen, the aortas were maintained in a −80°C freezer.

### 2.8 Polyacrylamide gel electrophoresis and Western blotting

Frozen aortic strips were washed with an ice‐cold acetone‐DTT solution 3–5 times before proceeding to homogenization to remove traces of TCA. Aortic strips were placed in Precellys cell lysis tubes containing 250 μL of homogenization buffer with protease and phosphatase inhibitor cocktail (Thermo Fischer Scientific Cat # 78440). The tubes were placed in a bead beater and 5 cycles (1 cycle: 1 min on‐30 s off—30 s on‐3 min off—2nd cycle) and were run at 5000 rpm with an interval of 3 min between each cycle at 4°C. Lysates were cleared by centrifugation at 12.5 K rpm for 10 min at 4°C. Protein concentration was estimated using a Bio‐Rad DC protein assay kit per the manufacturer's instructions.

Lysates were prepared by adding protein loading dye and the samples were heated by placing the Eppendorf tubes on a heat block for 3–5 min. Protein samples were separated by sodium dodecyl sulfate‐polyacrylamide gel electrophoresis (SDS‐PAGE) followed by semidry electrotransfer onto a nitrocellulose membrane (120 mAmp/ 90–180 min). Membranes were air dried for 30 min followed by blocking with Li‐CoR blocking buffer in Tris buffered saline (TBS) at room temperature for 1 h. Blots were incubated with primary antibodies as follows: ACTA2 (1:1000), CNN1 (1:500), CNN2 (1:500), CNN3 (1:500), p44/42ERK (1:200), Phospho p44/42ERK (1:250), Phospho MLC20 (1:250), Phospho MYPT1‐T850 (1:500), Phospho CPI17‐T38 (1:500), Vinculin (1:500) and GAPDH (1:10000) overnight at 4°C followed by washing with TBS‐Tween‐20 (0.05%) solution 5 times with an interval of 5 min. Blots were incubated with fluorescently labelled 2^0^ antibodies (1:10000) at room temperature for 90 min followed by above mentioned TBS‐Tween‐20 washings. Fluorescent signals were captured on a Li‐COR or Azure imaging system and processed in Microsoft PowerPoint for visualization.

### 2.9 Statistical analysis

Data were analysed by a student *t*‐test on Graphpad Prism 8.0 and a *p*‐Value of <0.05 was considered statistically significant.

## 3 RESULTS

### 3.1 CRISPR‐mediated single base substitution of a CArG box abolished CNN1 expression with minimal effects on the aortic transcriptome

**FIGURE 1:**
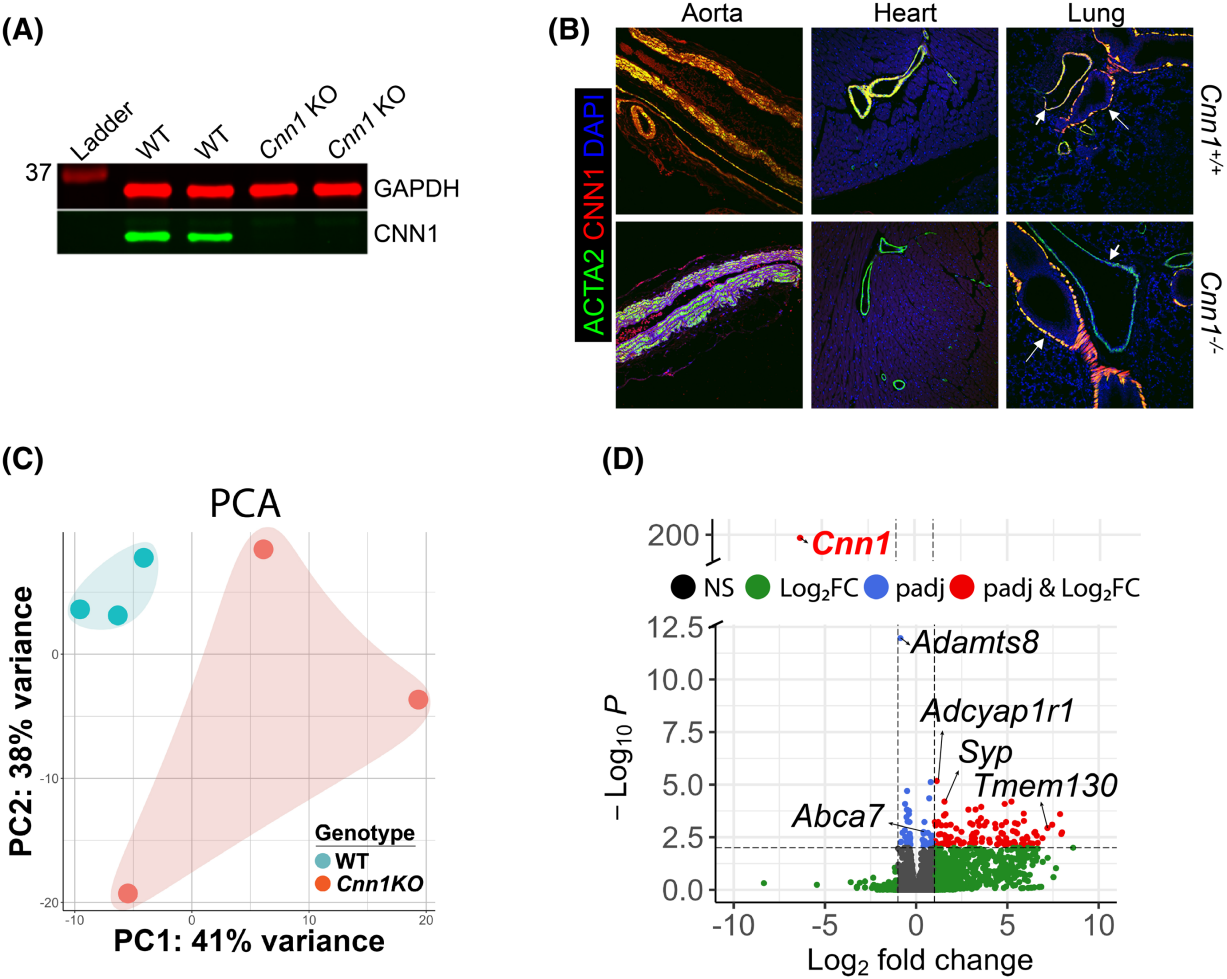
CNN1 expression and transcriptomic changes in *Cnn1* KO mouse aorta. (A) Western blot of indicated proteins in aorta. (B) Confocal immunofluorescence microscopy of indicated proteins colocalized in vessels of indicated tissues. Arrows, airway SMC; smaller arrows, vascular SMC. (C) PCA plot of WT and *Cnn1* KO aorta bulk RNA‐seq. *DeSeq2* was used to generate principal components. (D) Volcano plot of *Cnn1* KO Aorta with 30,822 genes plotted and 510 differentially expressed genes. Dotted lines indicate cutoffs for *p*‐adjusted value >0.01 (y‐axis) and Log_2_FC > |1.0| (x‐axis). Of note, *Cnn1* was the only gene to be both padj <0.01 and Log_2_FC < −1.0. Plot was generated with *EnhancedVolcano*.

Competitive EMSA revealed that a C > G transversion in the first base of the intronic CArG box was highly disruptive for SRF binding (Figure S1A). Accordingly, we used 3‐component CRISPR to engineer the same C > G transversion in mice (Figure S1B). A simple restriction digest of ear tag DNA distinguished this single‐base substitution from the wild‐type sequence (Figure S1C), and Sanger sequencing confirmed the C > G transversion with no detectable on‐target collateral damage (Figure S1D, data not shown). Quantitative RT‐PCR demonstrated barely detectable levels of *Cnn1* mRNA in the aorta of mice homozygous for the C > G transversion (hereafter referred as *Cnn1* KO); interestingly, there was only partial KO of *Cnn1* mRNA in visceral SMC‐containing tissues (Figure S2A). Western blot analysis showed an expected abrogation of CNN1 protein in the aorta of KO mice (Figure 1A). Confocal immunofluorescence imaging demonstrated a similar reduction in CNN1 protein in VSMCs of the aorta and smaller vessels of the heart and lung (Figure 1B). As with mRNA expression studies, confocal microscopy showed residual expression of CNN1 protein in several visceral SMC organs of *Cnn1* KO mice (Figure S2B). These results validate this *Cnn1* KO mouse, demonstrating a preferential loss of *Cnn1* mRNA and CNN1 protein expression in VSMCs.

To assess whether loss of CNN1 has any impact on steady‐state levels of global gene expression, we performed bulk RNA‐seq of the aorta, enriched for VSMCs. Principal component analysis (PCA) revealed a tight grouping of wild‐type aorta samples which were distinct from the more variable *Cnn1* KO aorta (Figure 1C). Using a false discovery rate of *p* < 0.01, DEseq2 analysis showed a massive reduction in *Cnn1* and only 146 differentially expressed genes (30 downregulated and 116 upregulated) (Figure 1D). These findings indicate that loss of CNN1 has only a small effect on the aortic SMC trancriptome, consistent with the known localization of CNN1 to the cytoplasm.^8^

### 3.2 CNN1 negatively regulates agonist‐induced ERK activation

**FIGURE 2:**
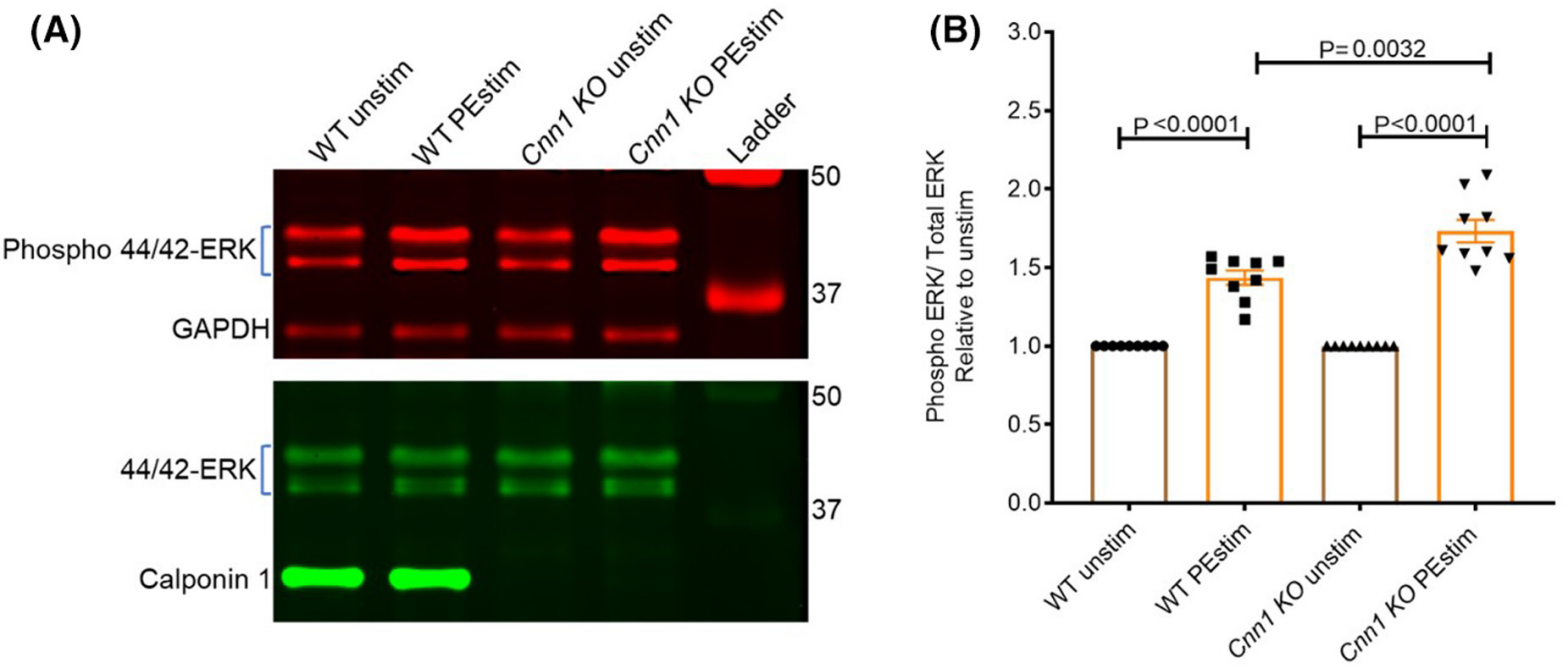
CNN1 inhibits agonist‐induced ERK activation. (A) Western blot demonstrating phospho ERK levels in wild type (WT) and *Cnn1* KO before and after stimulation with 10 μm phenylephrine (PE). (B) Graph representing increased ERK phosphorylation levels in the *Cnn1* KOs compared to WT. (WT, *n* = 9; *Cnn1* KO, *n* = 9). Phospho 44/42 ERK levels are normalized to total 44/42 ERK levels.

Our previous studies using aortic tissue from ferrets suggested a role for CNN1 as a scaffolding protein in mediating ERK activation.^15^ To test whether this function of CNN1 exists in mice, we measured agonist‐induced ERK phosphorylation in the aorta of WT and *Cnn1* KO mice, 15 min following stimulation with the alpha‐agonist phenylephrine (PE). Western blotting showed that ERK protein phosphorylation levels increased after PE treatment in both *Cnn1* KO and WT littermate control aortic strips with higher ERK phosphorylation levels in the KO compared to the WT (Figure 2). These results confirm and extend in mice our previous findings that CNN1 negatively regulates ERK activation in VSMCs.

### 3.3 *Cnn1* KO mice show reduced agonist‐induced stress and stiffness

**FIGURE 3:**
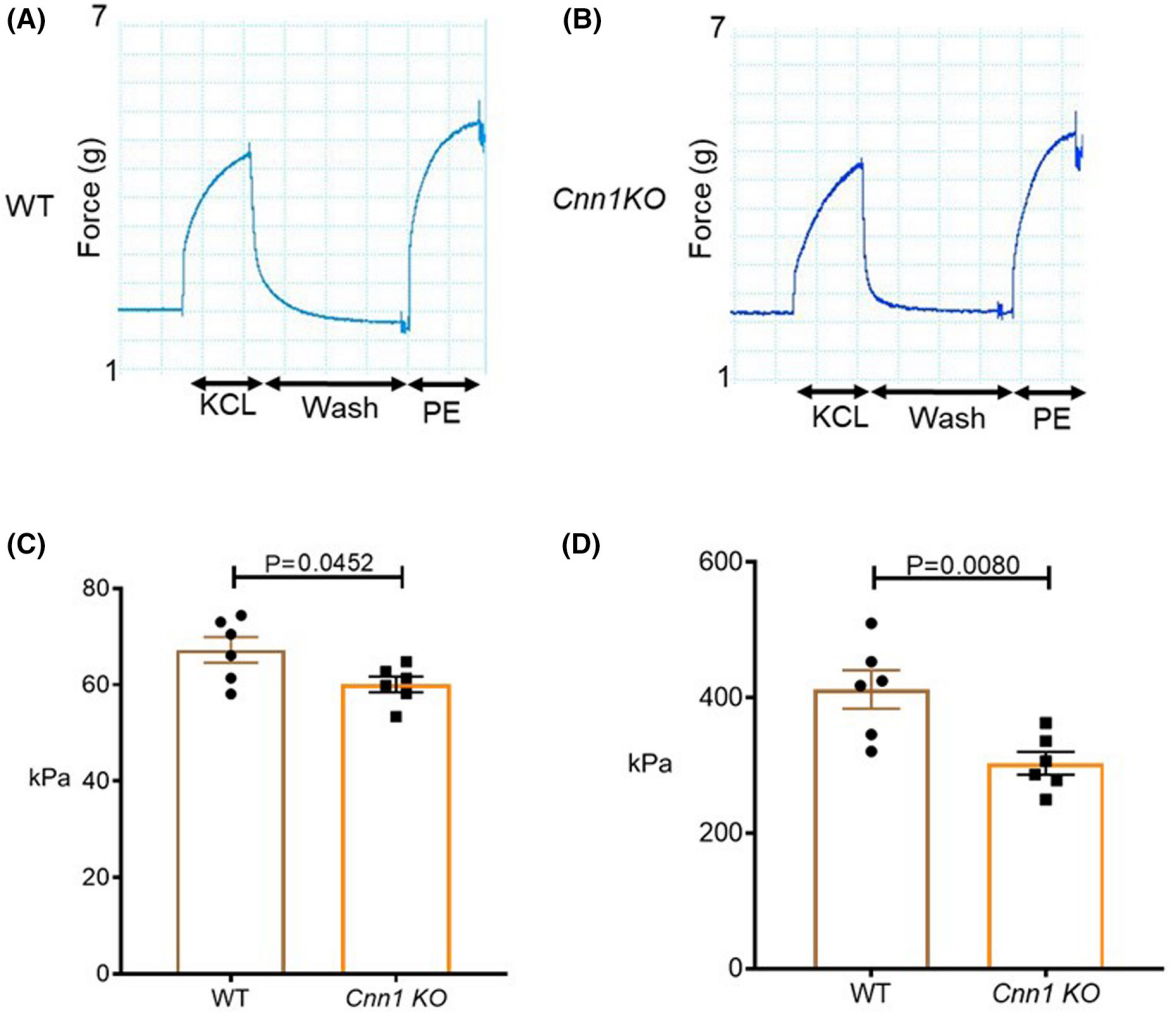
A reduction in agonist‐induced stress and stiffness in *Cnn1* KOs. (A,B) Myographs showing changes in force with different treatments in WT *and Cnn1* KO aortas. (C) Graph showing reduced agonist‐induced stress in the *Cnn1* KOs compared to WT. (D) Graph showing reduced agonist‐induced stiffness in the *Cnn1* KOs compared to WT. (kPa – Kilo Pascals). (WT, *n* = 6; *Cnn1* KO, *n* = 6).

VSMCs of the aorta play an important role in regulating the aortic luminal diameter to accommodate the pulsatile changes of blood through regulated events of contraction and relaxation.^22^, ^23^ In addition, VSMCs contribute to the stiffness of the aorta.^21^, ^24^ In previous in vitro studies, CNN1 was shown to be a negative regulator of smooth muscle contraction by inhibiting the actin‐activated Mg^+2^ ATPase activity of myosin.^10^ A recent study by Feng et al using aortas from *Cnn1* knockout mice showed a reduction in maximal force production by norepinephrine indicating a role for *Cnn1* as a positive regulator of VSMC contraction.^13^ In the present study, we explored the effect of *Cnn1* depletion on PE‐induced stress and stiffness of the mouse thoracic aorta. Results from our study demonstrate that depletion of *Cnn1* reduces PE‐induced stress and stiffness (Figure 3A–D). These and previously published results,^13^ support the notion of CNN1 playing an important role in the regulation of VSMC contraction.

### 3.4 *Cnn1* knockouts show prolonged Ca‐independent smooth muscle tone

**FIGURE 4:**
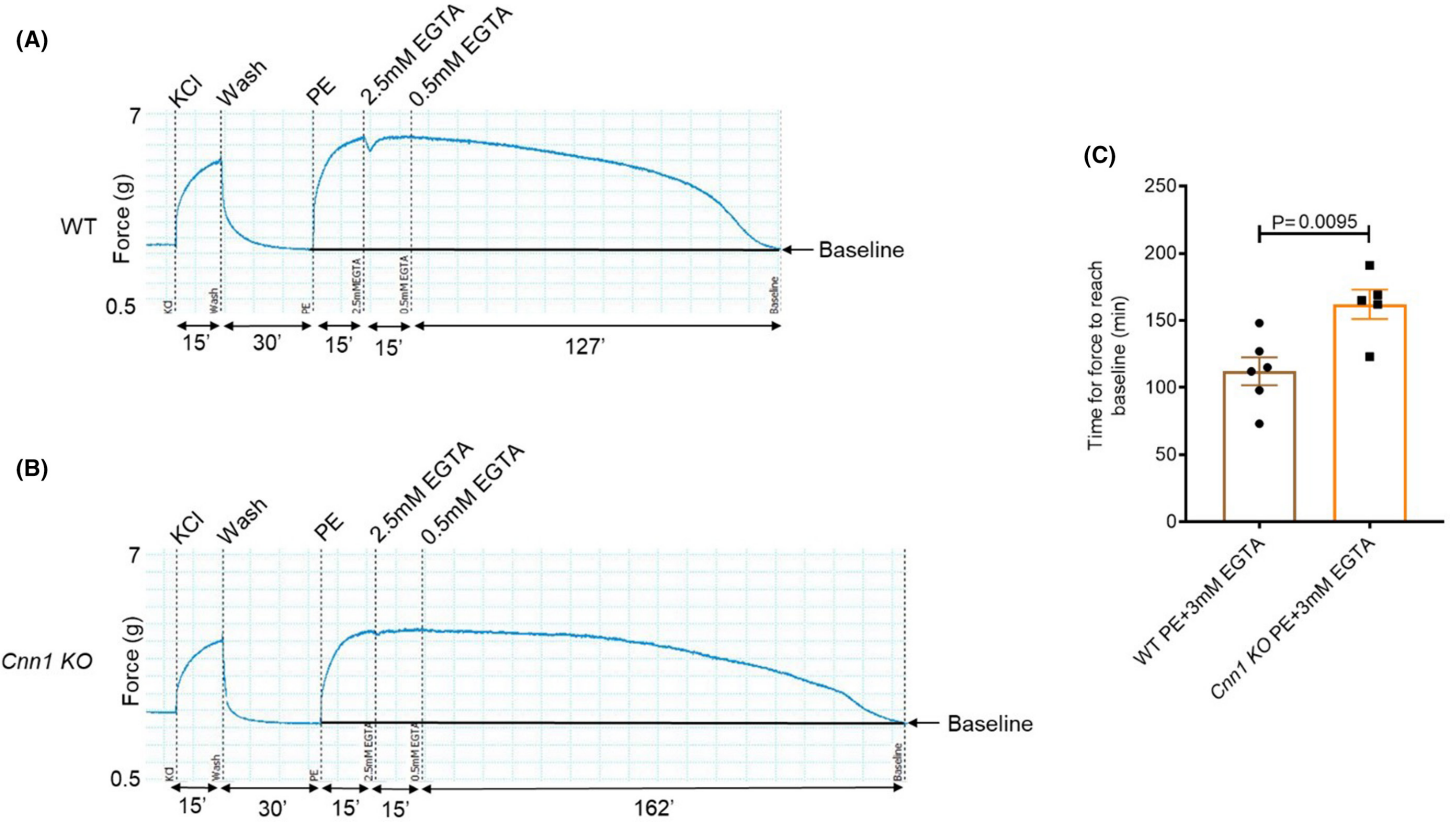
Increased duration of tone maintenance in *Cnn1* KO aortic strips under reduced calcium conditions. (A) Tone maintenance in the WT after adding EGTA. (B) Increased tone maintenance in the *Cnn1* KO compared to WT. (C) Graph representing the time for relaxation in wild type and *Cnn1* KO after adding EGTA to the buffer. (WT, *n* = 6; *Cnn1* KO, *n* = 5).

VSMCs have been shown to be capable of maintaining agonist‐induced smooth muscle tone under reduced intracellular calcium levels, a phenomenon termed calcium sensitization.^25^ In some studies, Rho kinase‐ or CPI‐17‐mediated inhibition of myosin light chain phosphatase (MLCP) was shown to be responsible for the calcium sensitization.^26^, ^27^, ^28^ On the contrary, our previous studies in ferret aorta showed that VSMCs can generate force in the absence of an increase in intracellular calcium concentration or changes in ERK‐dependent MLC phosphorylation levels.^15^ To test the role of CNN1 in the maintenance of smooth muscle tone under reduced calcium conditions, we treated aortic strips from both WT and *Cnn1* KO mice with PE for 15 min followed by the gradual addition of EGTA to a final concentration of 3 mM to reduce free calcium in the buffer. We then measured the time required for the force to reach baseline. The results showed that *Cnn1* KO aortic strips maintain tone longer than wild type aortic strips under these conditions (Figure 4A–C). These results suggest CNN1 negatively regulates calcium sensitization in VSMCs under conditions of reduced calcium.

### 3.5 CPI‐17 mediated inhibition of MLCP is responsible for calcium sensitization in *Cnn1* KOs

**FIGURE 5:**
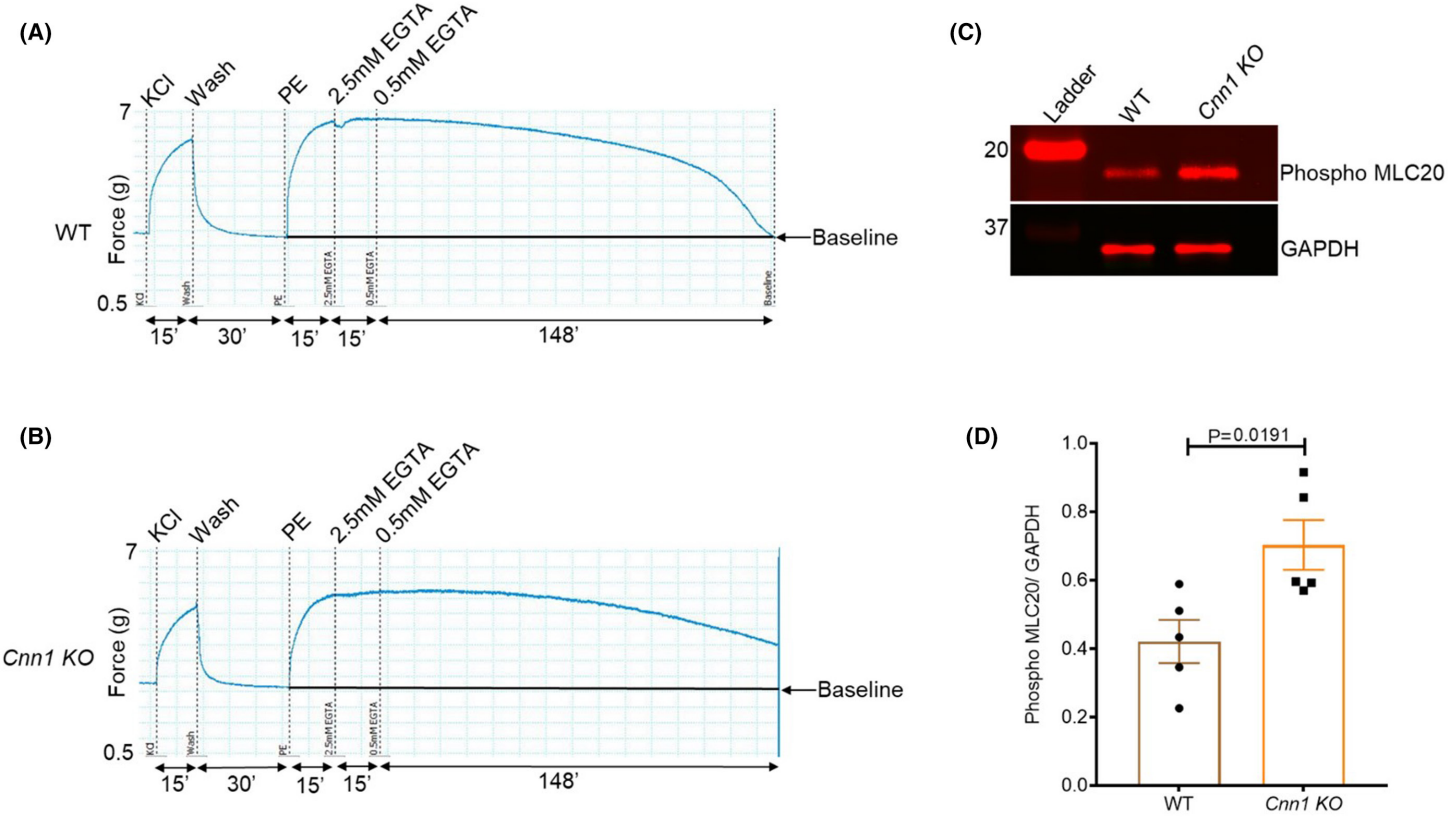
Smooth muscle tone is maintained longer due to increased myosin light chain phosphorylation in the *Cnn1* KOs. (A,B) Aortic strips were snap‐frozen at the same time in WT and *Cnn1* KOs to investigate the molecular mechanism responsible for increased tone maintenance in *Cnn1* KOs. (C) Myosin light chain phosphorylation (Phospho MLC20) in the *Cnn1* KO compared to the wild type. (D) Graph representing increased Phospho MLC20 levels in the *Cnn1* KO compared to WT. (WT, *n* = 5; *Cnn1* KO, *n* = 5).

To gain insight into the molecular mechanism responsible for increased maintenance of tone in *Cnn1* KO mice, we investigated the myosin light chain phosphorylation levels and found that MLC phosphorylation levels were higher in *Cnn1* KO aortas compared to wild type (Figure 5A–D). These results suggest that inhibition of myosin light chain phosphatase could be responsible for the observed MLC phosphorylation levels in the *Cnn1* KO.

**FIGURE 6:**
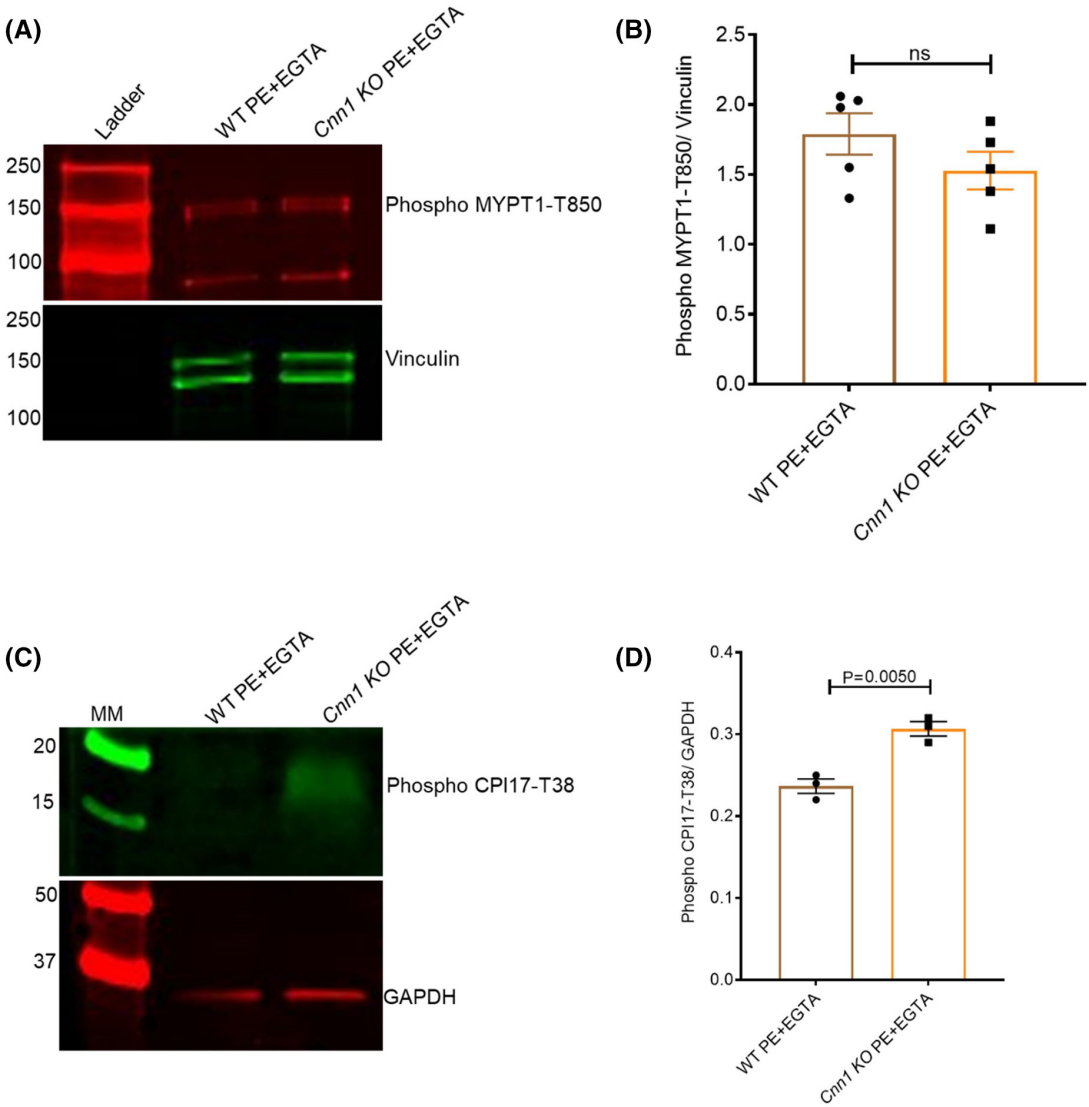
CPI17 inhibited the MLCP in *Cnn1* KOs. (A) Blot representing MYPT1‐T850 phosphorylation levels in WT and *Cnn1* KO aortas after treating with PE + EGTA. (B) Graph representing relative levels of phospho MYPT1‐T850 levels in WT and *Cnn1* KOs normalized to vinculin with no change observed between WT and *Cnn1* KO. (WT, *n* = 5; *Cnn1* KO, *n* = 5). (C) Blot representing Phospho CPI17‐T38 levels in WT and *Cnn1* KO aortas after treating with PE + EGTA. (D) Graph representing relative levels of phospho CPI17‐T38 levels in WT and *Cnn1* KOs normalized to GAPDH with more phospho CPI17 observed in *Cnn1* KO compared to WT. (WT, *n* = 3; *Cnn1* KO, *n* = 3).

Two different mechanisms have been reported to cause inhibition of MLCP in SMCs: (1) Rho‐kinase‐mediated phosphorylation of the MYPT1 subunit of MLCP inhibits MLCP targeting to myosin; and (2) Rho‐kinase or PKC‐mediated phosphorylation of CPI‐17, which binds to MLCP and inhibits its targeting to myosin.^29^, ^30^ Western blot results of MYPT1 and CPI‐17 phosphorylation levels in the WT and *Cnn1* KO aortic lysates treated with PE + EGTA showed that *Cnn1* KOs have more CPI17 phosphorylation compared to WT with no difference in MYPT1 phosphorylation levels between WT and *Cnn1* KOs (Figure 6A–D). These results are consistent with a mechanism involving inhibition of MLCP targeting by binding of CPI17.

## 4 DISCUSSION

**FIGURE 7:**
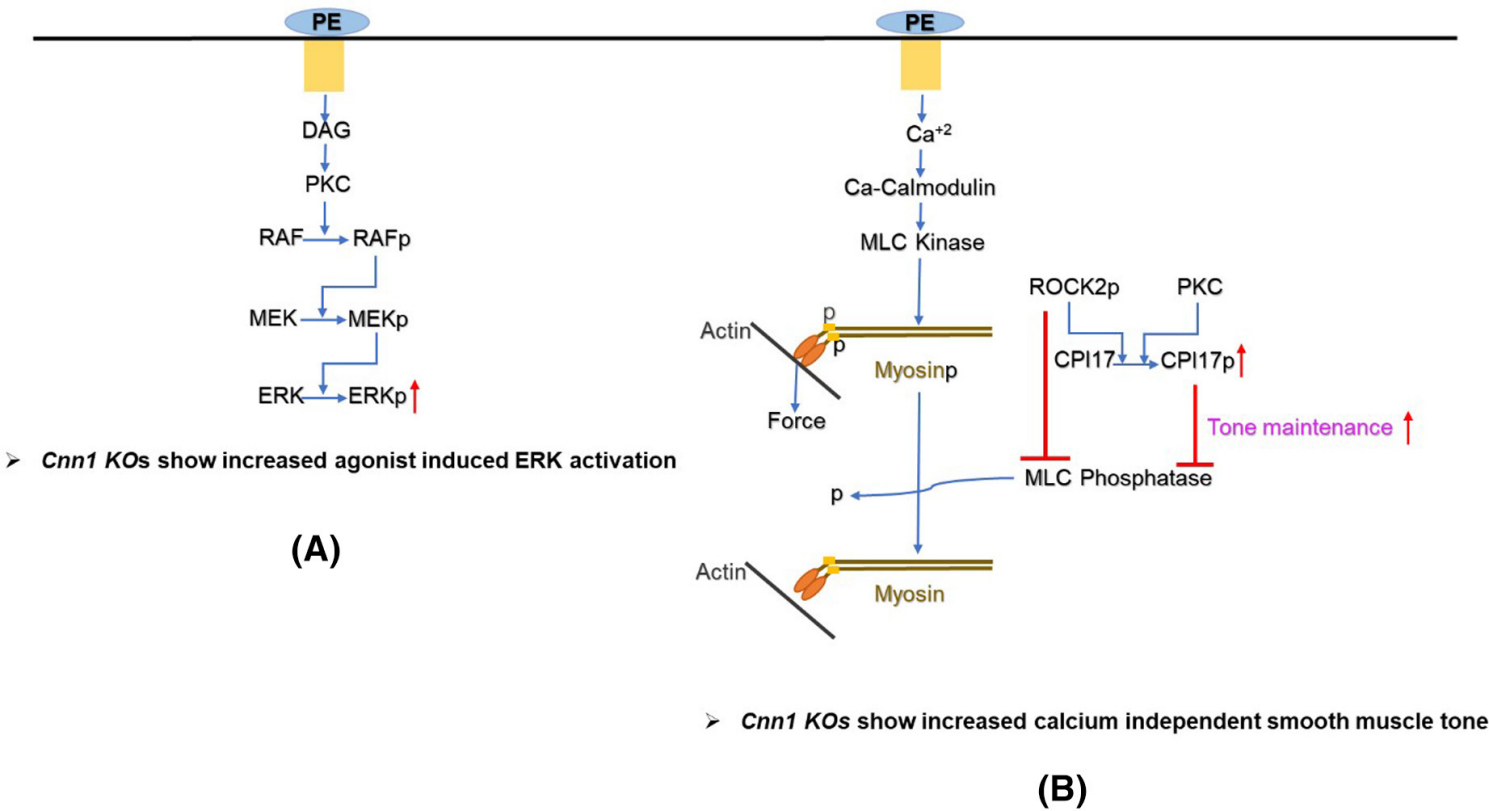
Schematic diagram illustrating the increased ERK activation and tone maintenance in the *Cnn1* KO aortas. (A) Binding of phenylephrine to alpha agonist receptor on smooth muscle cell surface results in the release of DAG (diacyl glycerol), and DAG‐mediated activation of PKC (protein kinase‐C). Once activated, PKC activates RAF (rapidly accelerated fibrosarcoma) which in turn activates the MEK (MAP kinase kinase). MEK activates ERK (extracellular signal regulated kinase). More ERK phosphorylation in the *Cnn1* KO indicates that CNN1 negatively regulates agonist ERK activation. (B) Phenylephrine increases the intracellular calcium ion concentration, activating MLCK (myosin light chain kinase) and subsequent myosin light phosphorylation. Phosphorylated myosin interacts with central actin filaments leading to crossbridge cycling and force production. MLCP (Myosin light chain phosphatase) inhibits smooth muscle contraction by removing the phosphate group from the myosin; however, inhibition of MLCP by Rho kinase‐mediated phosphorylation of myosin targeting subunit MYPT1 or inhibition of MLCP targeting to myosin by binding of phospho CPI17 (protein kinase‐C dependent phosphatase‐1 inhibitor of 17 kDa) leads to increased tone maintenance in the smooth muscle cells. *Cnn1* KO aortas showed an increase in calcium‐independent tone maintenance by CPI17‐mediated inhibition of MLCP.

The results of this study provide new insight into the function of CNN1 in vascular tissue. The extracellular signal regulated kinase was shown to play important roles in several intracellular processes in VSMCs which includes SMC contraction and proliferation. Previous studies from our laboratory showed that CNN1 interacts with both PKC and ERK post‐agonist stimulation. Further, CNN1 appears to play an important role in ERK activation by acting as a scaffolding protein bringing together the activators of ERK.^15^ The results presented in the current study show that depletion of CNN1 leads to increased agonist‐induced ERK activation in VSMCs (Figure 7). This finding suggests CNN1 in wild‐type mice negatively regulates agonist‐induced ERK activation.

Previous in vitro biochemical studies using purified calponin showed that CNN1 inhibits smooth muscle contraction by inhibiting actin‐activated Mg ATPase activity of myosin.^10^ However, later ex vivo studies using tissues from different animal models demonstrated a regulatory role of CNN1 in SMC contraction.^11^, ^12^ A recent study using aortas from a different *Cnn1* KO mouse than here demonstrated a reduction in maximal force production after norepinephrine treatment.^13^ In the present study, we also observed that the aortas of *Cnn1* KO mice showed a reduction in agonist‐induced stress confirming the role of CNN1 in SMC contraction. In addition, we also found that *Cnn1* KO aortas produce less agonist‐induced stiffness. Previous studies from our laboratory reported that SMCs of the aorta contribute to approximately 50% of the total aortic stiffness with the remaining coming from changes in the matrix.^21^

Smooth muscle cell contraction can be elicited by an increased intracellular calcium concentration resulting in the phosphorylation of myosin light chains (MLC) by calcium‐dependent myosin light chain kinase (MLCK). Phosphorylated myosin interacts with actin filaments leading to crossbridge cycling and smooth muscle contraction.^22^, ^31^ However, agonist‐induced smooth muscle tone has been shown to be maintained under reduced calcium achieved by an increase in the sensitivity of myofilaments called calcium sensitization. The calcium sensitization was shown to be achieved by the inhibition of myosin light chain phosphatase which removes the phosphate group from myosin light chains.^25^ Inhibition of MLCP allows the muscle to maintain the tone for a longer duration. Rho kinase‐dependent inhibition of MLCP has been shown to be responsible for calcium sensitization. In addition, Rho kinase or PKC‐mediated potentiation CPI17 was also shown to be responsible for the inhibition of MLCP.^27^, ^28^ Previous studies from our laboratory showed that calcium‐independent isoforms of PKC were responsible for force production in the absence of an increase in intracellular calcium levels. In addition, CNN1 was shown to interact with PKC in an agonist‐dependent manner; however, the role of this interaction was unclear.^15^ The current study's results indicate that CNN1 negatively regulates calcium sensitization as the *Cnn1* KO aortas maintain tone for a longer duration. As the extracellular calcium was removed by adding EGTA, the observed MLC phosphorylation levels in *Cnn1* KOs resulted from the inhibition of MLCP. Our further search for the mechanism responsible for the inhibition of MLCP resulted in the identification of increased phosphorylation of CPI17 which could maintain tone longer in *Cnn1* KOs by inhibiting the targeting of MLCP to myosin. The lack of increase in MYPT1 phosphorylation ruled out the involvement of Rho kinase‐mediated inhibition of MLCP. More CPI17 phosphorylation in the *Cnn1* KOs indicates the involvement of a calcium‐independent isoform of PKC whose activity could be inhibited by CNN1.

Calponin has three isoforms with CNN1 being specific to smooth muscle cells.^7^, ^8^ The absence of change in expression of the other two calponin isoforms, CNN2 and CNN3, in the *Cnn1* KO (Figure S3) indicates that the above observed effects are mediated by specific loss of CNN1 in VSMCs.

The studies herein were conducted with a new *Cnn1* KO mouse carrying a single base substitution in an intronic CArG box, a regulatory element that binds the ubiquitous transcription factor, SRF.^32^ This *Cnn1* KO differs from a previous KO wherein coding exons 5 and 6 were replaced with a neomycin cassette.^33^ The development of KO mice through regulatory element disruption leaves the coding sequence intact allowing for ease in genetic rescue experiments. For example, it should be possible to over‐ride the transcriptional silencing effect of regulatory element mutations through CRISPR activation.^34^ Remarkably, the single base transversion resulted in a true null *Cnn1* allele in VSMCs with near complete loss of CNN1 protein, but incomplete loss of CNN1 expression in visceral SMCs of the gastrointestinal tract and lung. Previously, we reported an identical single base transversion of a promoter CArG box in the SMC‐restricted *Tspan2* gene with similar abolition of *Tspan2* expression in VSMCs.^20^ These findings are noteworthy as they are the first to reveal the critical importance of a single base in driving full expression of two SMC‐restricted genes, *Cnn1* and *Tspan2*. Importantly, not all SRF/CArG‐dependent genes rely upon a single CArG box because aggressive inactivation of more than one CArG box in the *Lmod1* and *Srf* genes had minimal effects on each gene's expression in mice.^35^, ^36^ We assume that the latter two genes, which are essential for life, harbour additional regulatory elements to safeguard expression and tissue homeostasis whereas genes dispensable for life (*Cnn1* and *Tspan2*) lack such redundancy. It will be of interest to determine whether this trend holds with additional CArG‐dependent genes as well as other transcription factor‐binding sites.

## 5 CONCLUSION

A new VSMC‐restricted *Cnn1* KO mouse has been developed without disrupting the coding sequence. This model was used to extend previous studies conducted in ferrets, showing that CNN1 can act as an inhibitor of ERK activation and calcium sensitization.

## AUTHOR CONTRIBUTIONS

**Lova Prasadareddy Kajuluri:** Conceptualization (equal); data curation (equal); formal analysis (equal); investigation (equal); methodology (equal); project administration (equal); writing – original draft (equal); writing – review and editing (equal). **Qing Rex Lyu:** Conceptualization (equal); data curation (equal); investigation (equal); methodology (equal); resources (equal). **Jaser Doja:** Conceptualization (supporting); data curation (supporting); formal analysis (supporting); investigation (supporting); methodology (supporting). **Ajay Kumar:** Conceptualization (supporting); data curation (supporting); formal analysis (supporting); investigation (supporting); methodology (supporting). **Michael P. Wilson:** Conceptualization (supporting); data curation (supporting); formal analysis (supporting); investigation (supporting); methodology (supporting). **Samantha R. Sgrizzi:** Data curation (supporting); investigation (supporting); methodology (supporting). **Elika Rezaeimanesh:** Data curation (supporting); investigation (supporting); methodology (supporting). **Joseph M. Miano:** Conceptualization (equal); data curation (equal); funding acquisition (equal); methodology (equal); writing – review and editing (supporting). **Kathleen G. Morgan:** Conceptualization (lead); data curation (equal); formal analysis (equal); funding acquisition (lead); methodology (equal); project administration (lead).

## CONFLICT OF INTEREST STATEMENT

The authors declare they have no conflicts of interest.

## Supporting information


Figures S1–S3

## Data Availability

Data will be made available if contacted with a reasonable request.
