# Supplementary material for: Calponin 1 inhibits agonist‐induced ERK activation and decreases calcium sensitization in vascular smooth muscle
Source: J Cell Mol Med. 2023 Dec 26;28(1):e18025. doi: 10.1111/jcmm.18025 (PMC10805486; doi:10.1111/jcmm.18025)
Supplement: Supplementary file 1 — Figures S1–S3 [file JCMM-28-e18025-s001.zip › Figure captions.docx]

**Supplemental Fig** **1.** **Strategy for generating *Cnn1* KO mouse.** **A,** Competitive EMSA with radiolabeled CArG box incubated with 100x cold wild-type ssODN (CCTTATAAGG) or various single-base mutants of the wild-type sequence (substitutions in red). Note loss of SRF binding with cold wild-type (3rd lane from left), but not with C > G transversion in position 1 of CArG box (4th lane from left). **B,** 3-component CRISPR strategy for engineering C > G transversion in mice. The wild-type CArG box (blue line at top among three other CArG boxes, black lines) is depicted in blue letters with the partial ssODN repair template carrying a G (red) nucleotide at bottom. **C,** PCR product using primers flanking CArG box edit (arrows in B) was digested with SacII to obtain the banding pattern shown for each genotype. **D,** Sanger sequence confirmation of the installed G nucleotide in Cnn1−/− (KO). There were no detectable on-target insertions or deletions within the PCR product (not shown).

**Supplemental Figure** **2. Tissue expression of CNN1. A**, qRT-PCR of *Cnn1* in indicated tissues across aorta and various visceral SMC tissues. Results shown are of one animal/genotype with technical replicates. An independent mouse of each genotype showed similar results. **B** Confocal immunofluorescence microscopy of CNN1 in the indicated tissues of wild-type (top row) and *Cnn1* KO (bottom row) mice. Results were found in an independent mouse.

**Supplemental Figure** **3. CNN2 and CNN3 levels are unchanged in the *Cnn1* KO.** **A**, WT and *Cnn1* KO aortic lysates were probed with CNN2 and GAPDH antibodies. **B**, Graph representing the relative levels of CNN2 in WT and *Cnn1* KO normalized to GAPDH. (WT, n = 3; *Cnn1* KO, n = 3). **C**, WT and *Cnn1* KO aortic lysates probed with CNN3 and GAPDH antibodies. **D**, Graph representing relative levels of CNN3 in WT and *Cnn1* KO normalized to GAPDH. (WT, n = 6; *Cnn1* KO, n = 6).
