# Supplementary figures and images for: Calponin 1 inhibits agonist‐induced ERK activation and decreases calcium sensitization in vascular smooth muscle
Source: J Cell Mol Med. 2023 Dec 26;28(1):e18025. doi: 10.1111/jcmm.18025 (PMC10805486; doi:10.1111/jcmm.18025)

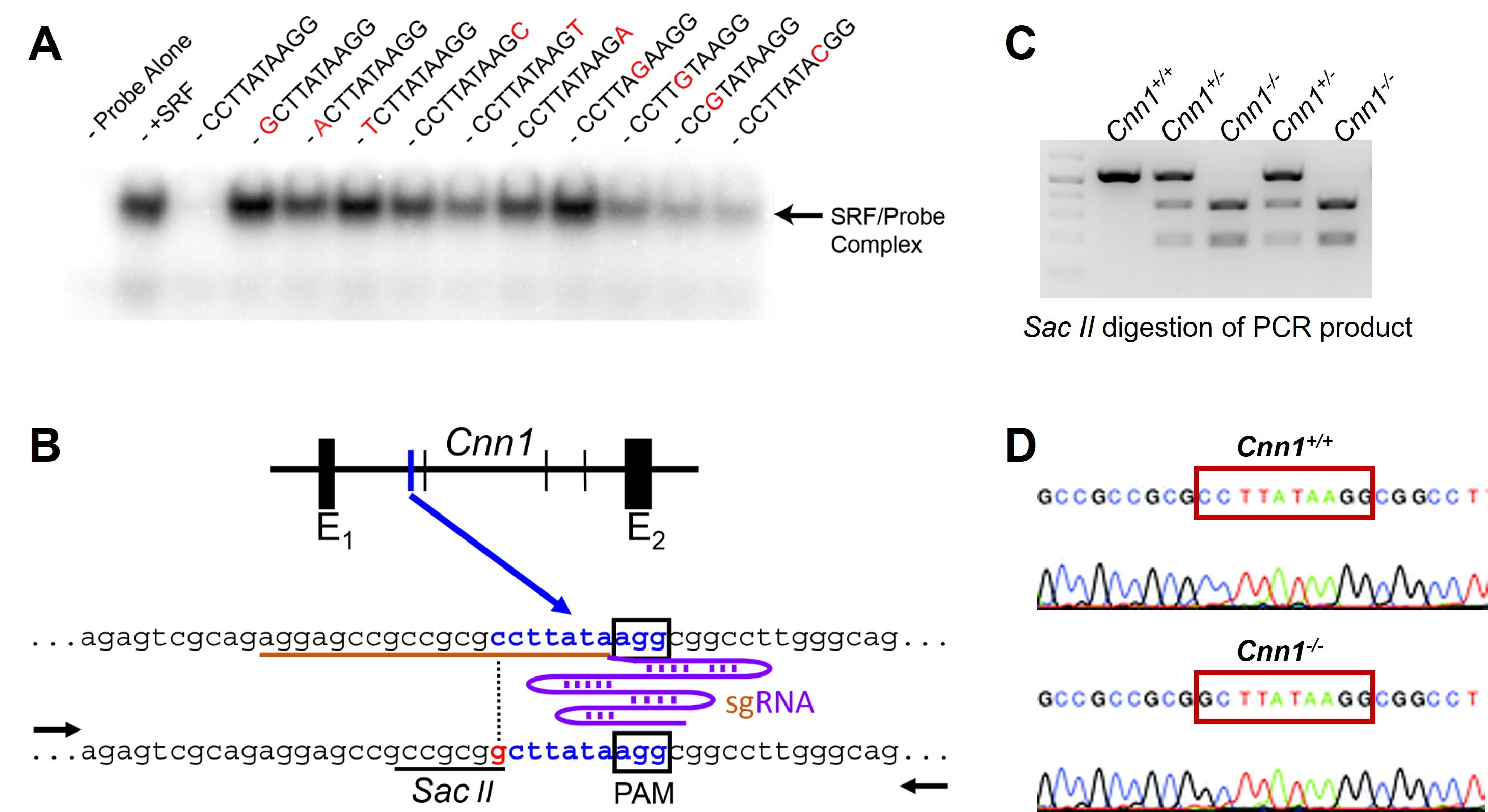

Supplement: Supplementary file 1 — Figures S1–S3 [file JCMM-28-e18025-s001.zip › jcmm18025-sup-0001-FigureS1.jpg]

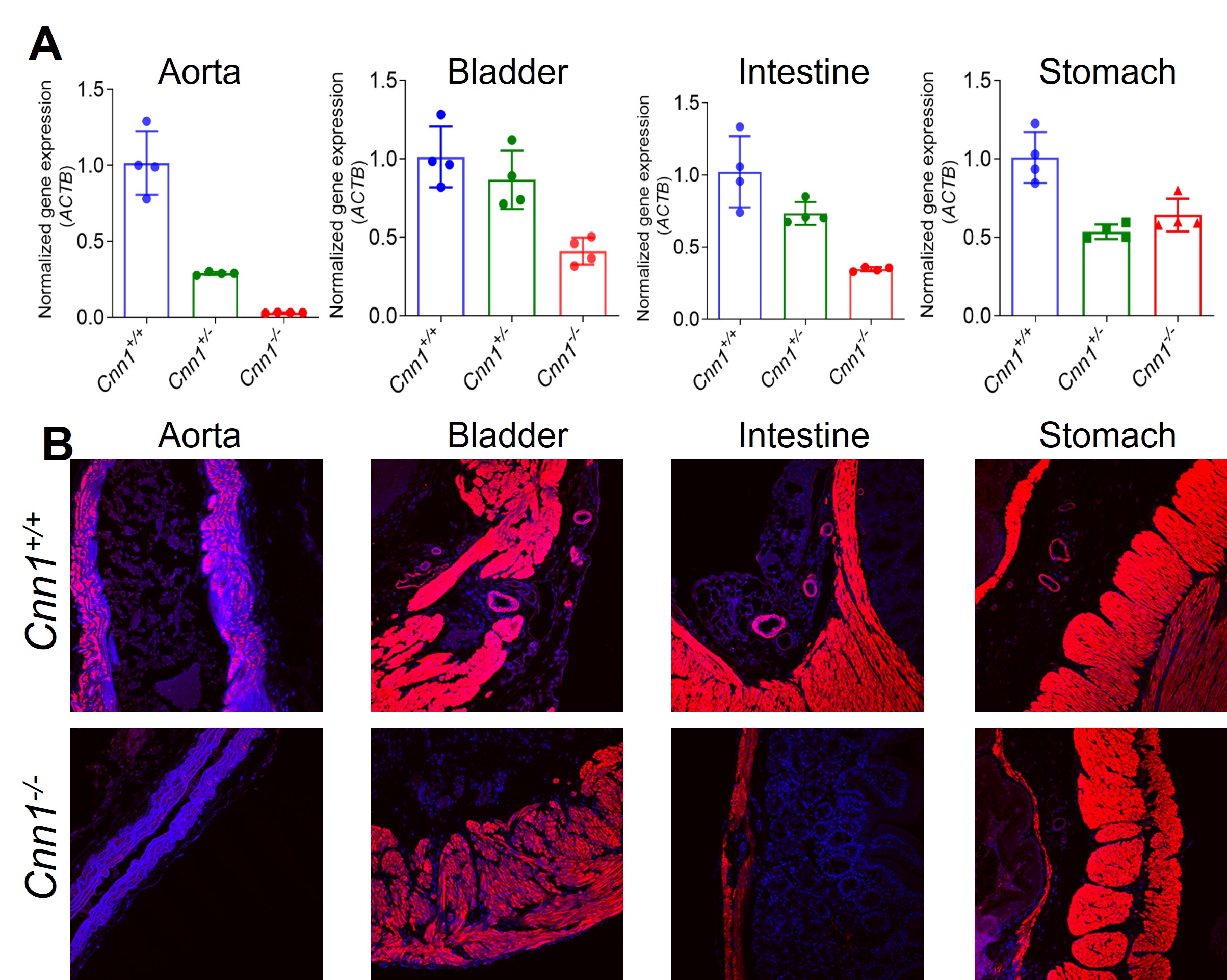

Supplement: Supplementary file 1 — Figures S1–S3 [file JCMM-28-e18025-s001.zip › jcmm18025-sup-0002-FigureS2.jpg]

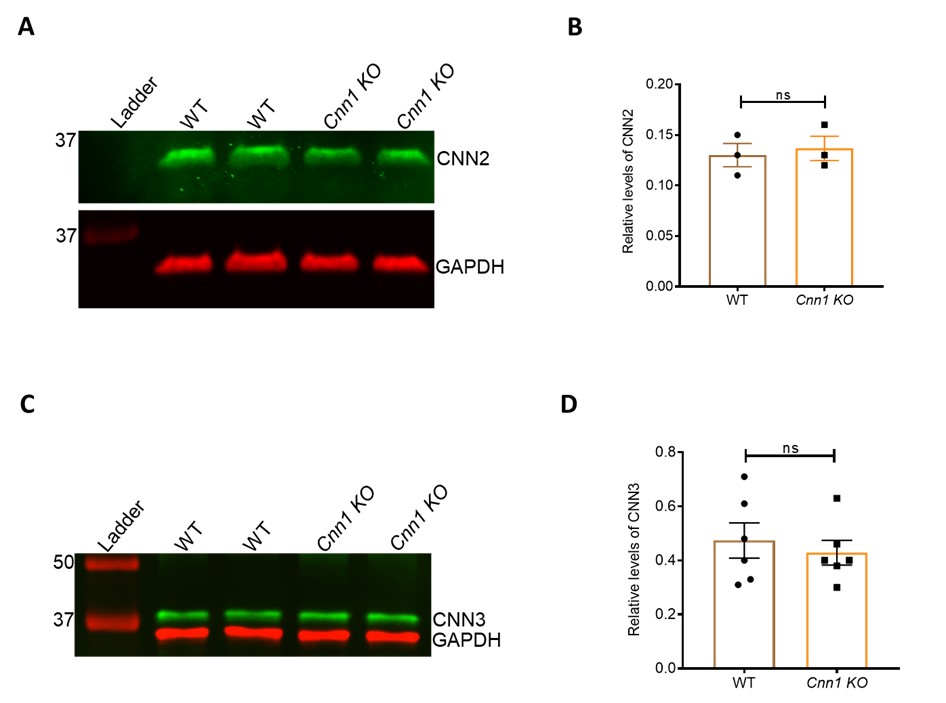

Supplement: Supplementary file 1 — Figures S1–S3 [file JCMM-28-e18025-s001.zip › jcmm18025-sup-0003-FigureS3.jpg]
